# Neuromyelitis optica spectrum disorder safely and successfully treated with satralizumab during pregnancy and breastfeeding: a case report

**DOI:** 10.3389/fneur.2023.1322412

**Published:** 2023-12-15

**Authors:** Takashi Yoshida, Osamu Watanabe, Miwa Nomura, Yusuke Yoshimoto, Yoshimitsu Maki, Hiroshi Takashima

**Affiliations:** ^1^Division of Neurology and Stroke, Kagoshima City Hospital, Kagoshima, Japan; ^2^Department of Neurology and Geriatrics, Kagoshima University Graduate School of Medical and Dental Science, Kagoshima, Japan

**Keywords:** neuromyelitis optica, IL-6, pregnancy, breastfeeding, satralizumab

## Abstract

**Background:**

Satralizumab, a monoclonal antibody that recognizes interleukin-6 receptors, is known to reduce the relapse rate in neuromyelitis optica spectrum disorder (NMOSD), but its safety during pregnancy has not been established. We present the case of an NMOSD patient who safely completed pregnancy, parturition, and breastfeeding under satralizumab treatment. Importantly, satralizumab transfer to umbilical cord blood, infant serum, or breast milk was not observed.

**Case presentation:**

A 37-year-old Japanese female developed anti-aquaporin 4 antibody-positive NMOSD with left optic neuritis. Despite responding to steroid and azathioprine therapy, she experienced moon face and weight gain and desired the prompt reduction of the steroid dosage. She also wanted to conceive a child with a safe and preferably early pregnancy and parturition. Because pregnancy and parturition after the onset of NMOSD elevate the risk of relapse and miscarriage, treatment with satralizumab was initiated with the patient's consent. She experienced normal parturition and continued with satralizumab, steroid, and azathioprine treatments while breastfeeding without experiencing any relapses. Concentrations of satralizumab in the umbilical cord blood, infant serum, and breast milk were below the detection sensitivity.

**Conclusion:**

These findings suggest that satralizumab may be safe and effective for the perinatal management of NMOSD, especially when there are concerns about continuing pregnancy and the risk of relapse after parturition.

## Introduction

Neuromyelitis optica spectrum disorder (NMOSD) is an inflammatory disease of the central nervous system primarily influenced by anti-aquaporin (AQP) 4 antibodies. These antibodies target astrocytes, causing severe damage to the optic nerve, spinal cord, and brain (1). Interleukin-6 (IL-6) has significant roles in the pathophysiology of NMOSD, exhibiting multiple functions such as stimulating plasmablasts and inducing the production of anti-AQP4 antibodies (2), reducing the expression of claudin-5 in vascular endothelial cells, increasing the permeability of the blood–brain barrier (3), and promoting the differentiation and activation of inflammatory T lymphocytes (4).

Satralizumab is a novel humanized immunoglobulin (Ig)G2 anti-IL-6 receptor monoclonal recycling antibody that is used as a treatment for NMOSD patients positive for anti-AQP4 antibodies. By inhibiting the binding of IL-6 to its receptor, satralizumab is expected to have four effects: reducing the production of anti-AQP4 antibodies, suppressing the differentiation and activation of inflammatory T cells, decreasing the permeability of the blood–brain barrier, and inhibiting astrocyte damage. Treatment with satralizumab was shown to significantly reduce the annualized relapse rate (ARR) in patients with NMOSD (5, 6), but its safety in pregnant females and fetuses has not been adequately established. Here, we report the case of an NMOSD patient who tested positive for anti-AQP4 antibodies and who completed pregnancy, parturition, and breastfeeding without any issues while continuing treatment with satralizumab, steroid, and azathioprine. Importantly, we observed that there was no evidence of satralizumab transfer to umbilical cord blood, infant serum, or breast milk.

## Case presentation

A 37-year-old Japanese female, generally in good health, began to experience visual difficulties in the lower part of her left eye, which gradually spread to her entire field of vision, resulting in blurred vision in her left eye. On day 5 after onset, she visited a local ophthalmologist, who noted significant vision loss in her left eye and promptly referred her to our hospital on the same day. At the time of onset, she was engaged and had plans to get married the following month. At the initial examination, her left visual acuity was significantly decreased, with unaided visual acuity measuring counting fingers at a distance of 50 cm and corrected visual acuity measuring 0.02. The critical fusion frequency could not be measured in the left eye. The left eye exhibited a positive relative afferent pupillary defect, and visual evoked potentials revealed the absence of a P100 response upon stimulation of the left eye. No significant abnormalities were found in the blood or cerebrospinal fluid, and head and spinal cord magnetic resonance imaging revealed no apparent lesions other than left optic neuritis. On day 12, she tested positive for anti-AQP4 antibody by enzyme-linked immunosorbent assay, leading to a diagnosis of NMOSD.

The administration of 1,000 mg/day intravenous methylprednisolone for 3 days starting on day 5 led to an improvement in the condition of her left visual field blindness. As post-therapy, 50 mg/day of oral prednisolone (PSL) was initiated on day 8 and gradually tapered off. However, because visual field impairment persisted thereafter, intravenous methylprednisolone at a daily dose of 1,000 mg for 3 days was repeated three more times over the course of the following 2 months, resulting in a total of four treatments. In addition, azathioprine was initiated and gradually increased up to 75 mg/day. Subsequently, there was a gradual improvement in her left visual acuity, but it remained insufficient; therefore, intravenous immunoglobulin therapy was initiated once a month starting on day 31. Thereafter, with no relapse, her left corrected visual acuity improved from 0.02 to 0.60, enabling her to decrease the dosage of PSL to 20 mg/day on day 85. However, moon face and weight gain became evident because of the prolonged use of steroids, and she expressed a desire to reduce the steroid dosage as soon as possible. She also wanted to conceive a child and have a safe and preferably early pregnancy and parturition.

Because pregnancy and parturition after the onset of NMOSD elevate the risk of relapse and miscarriage (further details will be provided later), we considered introducing satralizumab. After being informed that the safety of satralizumab in pregnant females had not been established and that there might be a risk of transfer to the fetus, the patient requested treatment with satralizumab, which was initiated on day 110.

After the administration of satralizumab, no specific adverse events were observed, and a positive pregnancy test was detected on day 140. Considering the risks of gestational diabetes and hypertension, discontinuing steroids was also contemplated. However, following consultations with our obstetrics and gynecology department, it was determined that the risks of disease relapse and miscarriage associated with discontinuing PSL and azathioprine were greater. Ultimately, the patient expressed a desire to continue the medication, so the decision was made to continue with PSL and azathioprine. During pregnancy, the regular monitoring of parameters including blood sugar levels and blood pressure was conducted, and all values remained within the normal range.

**Figure 1:**
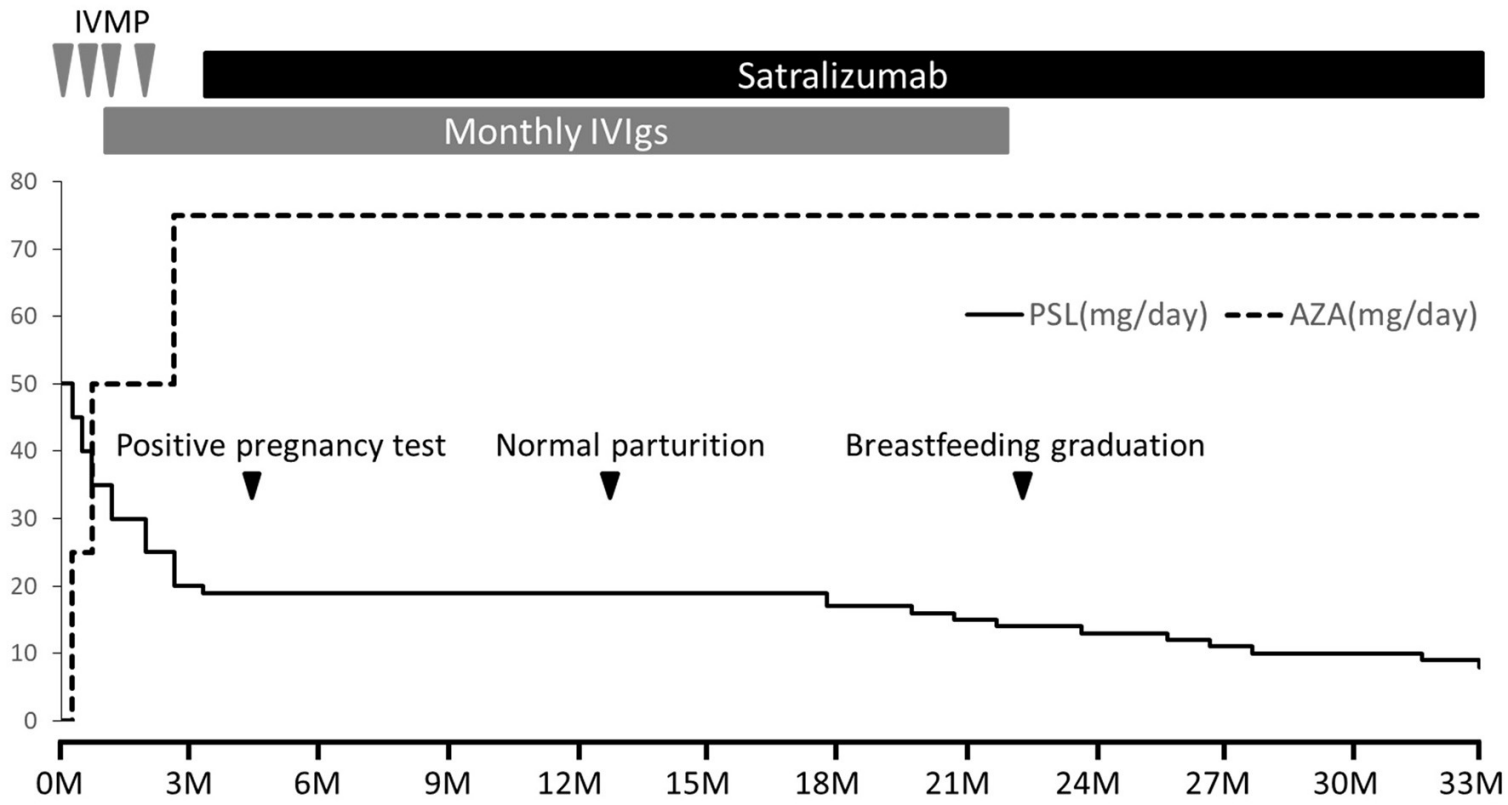
Clinical course after admission. NMOSD was managed without relapse using four rounds of intravenous methylprednisolone therapy (IVMP, 1,000 mg/day for 3 days), monthly intravenous immunoglobulin therapy (IVIg, 400 mg/kg/day for 5 days), oral prednisolone (PSL), and azathioprine (AZA). However, the patient experienced steroid-related side effects including moon face and weight gain, prompting early PSL tapering. With a desire for pregnancy and prioritizing disease stability, satralizumab was introduced from day 110 after onset (initial dose of 120 mg subcutaneously, followed by injections after 2 and 4 weeks, and subsequently every 4 weeks). A positive pregnancy test occurred on day 140, and normal parturition took place on day 380. Both mother and infant remained healthy, with normal growth and development. Breastfeeding continued until 10 months after birth. Over 2 years and 6 months, a total of 34 doses of satralizumab were administered without relapse, and the PSL dosage was reduced to 8 mg/day.

She received regular satralizumab doses and gave birth to a female infant weighing 3,200 g by normal vaginal parturition on day 380, at 39 weeks and 5 days of gestation. Both the mother and infant progressed smoothly without any abnormalities, and breastfeeding was continued until 10 months after birth. Throughout this period, the infant did not experience any serious infections and did not show any developmental delays. Anti-AQP4 antibody in the patient's serum was confirmed negative 20 months after onset. A total of 34 doses of satralizumab were administered over 2 years and 6 months, during which there were no relapses, and the PSL dosage was reduced to 8 mg/day (Figure 1).

The concentrations of satralizumab were measured in the maternal serum 21 days before parturition, in the maternal serum and umbilical cord blood immediately after parturition, and in the maternal serum, breast milk, and infant serum 5 and 26 days after parturition (Table 1). On the day of parturition (21 days after satralizumab administration), the concentration in the maternal serum was 9.59 μg/ml, and the umbilical cord blood concentration was below the detection sensitivity (< 0.200 μg/ml). Additionally, the maternal serum concentration 5 days after parturition (26 days after satralizumab administration) was 4.69 μg/ml, and the maternal serum concentration 26 days after parturition (19 days after satralizumab administration) was 10.3 μg/ml, whereas the concentrations in breast milk and infant serum were below the detection sensitivity.

**Table 1:** Concentrations of satralizumab (SAT) in the perinatal to postpartum period.

Days after parturition	−21	0	5	26
Days after SAT administration	0	21	26	19
**Maternal related (μg/ml)**				
Serum	7.87	9.59	4.69	10.3
Breast milk			< 0.200	< 0.200
**Infant related (μg/ml)**				
Umbilical cord blood		< 0.200		
Serum			< 0.200	< 0.200

## Discussion and conclusions

We present a patient with anti-AQP4 antibody-positive NMOSD presenting with left optic neuritis who experienced normal parturition under satralizumab, steroid, and azathioprine treatment without any abnormalities developing in the mother or infant. There were no relapses, and the concentrations of satralizumab in the umbilical cord blood, infant serum, and breast milk were below the detection sensitivity.

Pregnancy and parturition after the onset of NMOSD are associated with an increased relapse rate, with the highest ARR occurring during the 3 months after parturition (7–9). Furthermore, pregnancies following the onset of NMOSD have a higher odds ratio for miscarriage, and NMOSD patients who experienced miscarriages tended to have a higher ARR (10). These findings indicate elevated disease activity and highlight the necessity for more intensive treatment of young females with NMOSD who wish to conceive, focusing on disease activity suppression, relapse prevention, and maintenance of fertility.

Treatment with satralizumab significantly reduced the ARR of NMOSD (5, 6), but its safety during pregnancy, parturition, and breastfeeding has not been established definitively. In animal studies, satralizumab was shown to pass through the placenta and be transferred to infants and breast milk. To the best of our knowledge, no clinical studies have specifically investigated the placental passage and transfer of satralizumab to infants and breast milk. Satralizumab is a humanized anti-IL-6 receptor neutralizing antibody that was designed by modifying the amino acid sequence of tocilizumab, an IgG1 antibody, to extend its plasma half-life, resulting in a modified IgG2 form. A case report examining the safety of tocilizumab during pregnancy confirmed that approximately one-seventh of the maternal serum concentration of tocilizumab was transferred to the umbilical cord blood on the day of parturition (11). Because there were no issues with the infant's growth and development, it was concluded that treatment with tocilizumab could be managed during pregnancy (11). The lower placental transfer of IgG2 compared with IgG1 might be a contributing factor to the lack of transfer of satralizumab to the infant in this case. Additionally, because the lower limit of quantitation in this measurement was 0.200 μg/ml, there is still a possibility that transfer occurred, which might be confirmed using more precise measurement systems. A study limitation was that the satralizumab pharmacokinetic assay was validated for serum only. Moreover, in this case, the concurrent therapy with PSL and azathioprine was continued alongside satralizumab. Therefore, it is challenging to definitively determine whether the suppression of NMOSD relapse was attributable to satralizumab alone, to PSL and azathioprine, or to their combination. Further comparative studies are desired to address this aspect. Regardless, the transfer of satralizumab to infant serum and breast milk is extremely low, suggesting that it can be safely administered during pregnancy and breastfeeding.

In conclusion, we report a patient with NMOSD who completed pregnancy, parturition, and breastfeeding under satralizumab, steroid, and azathioprine treatment without relapse for 2 years and 6 months. The concentrations of satralizumab in the umbilical cord blood, infant serum, and breast milk were below the detection sensitivity. Thus, satralizumab may be safe and effective for the perinatal management of NMOSD, especially when there are concerns about continuing pregnancy and the risk of relapse after parturition.

## Data availability statement

The original contributions presented in the study are included in the article/supplementary material, further inquiries can be directed to the corresponding author.

## Ethics statement

Ethical review and approval was not required for the studies involving humans in accordance with the local legislation and institutional requirements. The participants provided their written informed consent to participate in this study. Written informed consent was obtained from the individual(s) for the publication of any potentially identifiable images or data included in this article.

## Author contributions

TY: Data curation, Investigation, Writing – original draft, Writing – review & editing. OW: Supervision, Writing – review & editing. MN: Data curation, Investigation, Writing – review & editing. YY: Data curation, Investigation, Writing – review & editing. YM: Data curation, Investigation, Writing – review & editing. HT: Supervision, Writing – review & editing.
